# Metformin improves diastolic function in an HFpEF-like mouse model by increasing titin compliance

**DOI:** 10.1085/jgp.201812259

**Published:** 2019-01-07

**Authors:** Rebecca E. Slater, Joshua G. Strom, Mei Methawasin, Martin Liss, Michael Gotthardt, Nancy Sweitzer, Henk L. Granzier

**Affiliations:** 1 Department of Cellular and Molecular Medicine, University of Arizona, Tucson, AZ; 2 Neuromuscular and Cardiovascular Cell Biology, Max Delbrück Center for Molecular Medicine, Berlin, Germany; 3 German Center for Cardiovascular Research, Partner Site Berlin, Berlin, Germany; 4 Sarver Heart Center, College of Medicine, University of Arizona, Tucson, AZ

## Abstract

Heart failure with preserved ejection fraction (HFpEF) is a complex syndrome characterized by a preserved ejection fraction but increased diastolic stiffness and abnormalities of filling. Although the prevalence of HFpEF is high and continues to rise, no effective therapies exist; however, the diabetic drug metformin has been associated with improved diastolic function in diabetic patients. Here we determine the therapeutic potential of metformin for improving diastolic function in a mouse model with HFpEF-like symptoms. We combine transverse aortic constriction (TAC) surgery with deoxycorticosterone acetate (DOCA) supplementation to obtain a mouse model with increased diastolic stiffness and exercise intolerance. Echocardiography and pressure–volume analysis reveal that providing metformin to TAC/DOCA mice improves diastolic function in the left ventricular (LV) chamber. Muscle mechanics show that metformin lowers passive stiffness of the LV wall muscle. Concomitant with this improvement in diastolic function, metformin-treated TAC/DOCA mice also demonstrate preserved exercise capacity. No metformin effects are seen in sham operated mice. Extraction experiments on skinned ventricular muscle strips show that the metformin-induced reduction of passive stiffness in TAC/DOCA mice is due to an increase in titin compliance. Using phospho-site-specific antibodies, we assay the phosphorylation of titin’s PEVK and N2B spring elements. Metformin-treated mice have unaltered PEVK phosphorylation but increased phosphorylation of PKA sites in the N2B element, a change which has previously been shown to lower titin’s stiffness. Consistent with this result, experiments with a mouse model deficient in the N2B element reveal that the beneficial effect of metformin on LV chamber and muscle stiffness requires the presence of the N2B element. We conclude that metformin offers therapeutic benefit during HFpEF by lowering titin-based passive stiffness.

## Abstract (precis)

Heart failure with preserved ejection fraction (HFpEF) is a syndrome characterized by increased diastolic stiffness, for which effective therapies are lacking. Slater et al. show that metformin lowers titin-based passive stiffness in an HFpEF mouse model and may therefore be of therapeutic benefit.

## Introduction

Heart failure with preserved ejection fraction (HFpEF) is a complex syndrome with preserved systolic function but diastolic stiffening that contributes to filling abnormalities. The culmination of these changes is left ventricular (LV) hypertrophy, decreased cardiac reserve, and pulmonary edema (Norman et al., 2011; Borlaug, 2014). The prevalence of HFpEF is increasing and is predicted to exceed 8% of persons older than 65 by the year 2020 (van Heerebeek and Paulus, 2016). Studies on myocardial biopsies from HFpEF patients have revealed alterations in myocardial structure, function, and signaling. These alterations include cardiomyocyte hypertrophy and interstitial fibrosis, increased cardiomyocyte stiffness, and down-regulation of cyclic guanosine monophosphate–protein kinase G (cGMP-PKG; Yusuf et al., 2003; Linke and Hamdani, 2014; van Heerebeek and Paulus, 2016).

Muscle stiffness is determined by both the elastic protein titin and the extracellular matrix (ECM) with the former dominating at physiological sarcomere lengths (Granzier and Labeit, 2006; Linke and Hamdani, 2014). A recent study measured the ECM- and titin-based passive tension in myocardial biopsies from HFpEF patients and found increased stiffness in both ECM and titin (Zile et al., 2015). Titin-based stiffness is determined by relative expression of the stiffer (N2B) and more compliant (N2BA) isoforms and by various posttranslation modifications including phosphorylation of spring elements within titin (LeWinter and Granzier, 2014). Phosphorylation of the cardiac-specific N2B spring element of titin by PKA and PKG is known to reduce cardiomyocyte stiffness, while PKC phosphorylation of the PEVK spring element increases stiffness (Hidalgo and Granzier, 2013). Increased phosphorylation of PKC sites and decreased phosphorylation of PKA sites are present in HFpEF patients, consistent with the increased measured stiffness of titin (Zile et al., 2015).

Unlike heart failure with reduced ejection fraction, pharmacological approaches to treating HFpEF do not currently exist, and earlier clinical HFpEF trials have been plagued by neutral results (Yusuf et al., 2003; Massie et al., 2008; Redfield et al., 2012, 2013). For example, the RELAX trail was designed to enhance PKG activity through PDE-5 inhibition, but clinical outcomes in the treated group were similar to placebo (Redfield et al., 2012, 2013). Given the lack of effective HFpEF therapies, additional therapeutic approaches are of great interest. Our present work on metformin, a drug commonly used for treatment of type 2 diabetes, was motivated by the finding in patients with diabetes that metformin is associated with improved LV diastolic function (Andersson et al., 2010). Additionally, the ongoing clinical trial Metformin in the Diastolic Dysfunction of Metabolic Syndrome investigates whether the effect of adding metformin to standard treatment of patients with metabolic syndrome ameliorates diastolic dysfunction (Ladeiras-Lopes et al., 2014). To evaluate whether metformin might have a beneficial effect on diastolic function in HFpEF, we studied metformin in a mouse model with HFpEF-like characteristics. The HFpEF was induced through transverse aortic constriction (TAC) surgery to increase afterload with concomitant mineralocorticoid supplementation (deoxycorticosterone acetate [DOCA]). This is a mouse model with HFpEF characteristics such as increased LV stiffness (both cardiomyocyte and ECM derived), diastolic dysfunction, concentric hypertrophy, preserved ejection fraction, and exercise intolerance (Mohammed et al., 2010; Methawasin et al., 2016). Additionally, we studied a mouse model in which we had deleted the N2B element within cardiac titin (N2B KO) resulting in a restrictive filling pattern and increased diastolic wall stress (Radke et al., 2007). We aimed to determine whether diastolic function can be improved in these models through treatment with metformin. Our findings establish that metformin improves diastolic function in the TAC/DOCA model, at both the LV chamber and isolated muscle levels. On the molecular level, metformin increases phosphorylation levels of PKA sites within titin’s N2B element as determined with phospho-site-specific antibodies.

The notion that metformin works through titin’s N2B element is supported by the lack of a metformin effect in a mouse model that is deficient in the N2B element.

## Materials and methods

### Mice and TAC/DOCA procedure

Mice on a C57BL/6J background were used for all experiments. We used both WT control animals and N2B KO mice that had the N2B element (exon 49) removed as described (Radke et al., 2007). In the N2B KO the deletion of exon 49 leads to splicing of exons 48–50, removing 2,646 bp (882 aa), which maintains the open reading frame. Mice were studied either at baseline or following either sham or TAC/DOCA surgery. The TAC/DOCA mice underwent surgery and pellet implantation as described below. Minimally invasive TAC was performed on 10-wk-old male mice as described previously with modification (Methawasin et al., 2016). In brief, mice were anesthetized using a single injection of ketamine and xylazine (120 and 12 mg/kg, intraperitoneal) and a 5-mm horizontal incision was made at the first left intercostal space. The thymus was temporarily retracted to visualize the aortic arch, and a 7–0 silk suture was passed under the aorta between the right innominate and left carotid arteries. The suture was ligated around a blunted 27-gauge needle, and the needle was quickly removed. The chest wall and skin were closed. An additional incision was made in the right flank of the animal, and a subcutaneous pocket was created by blunt dissection. A DOCA (50 mg per pellet, 21-d release) pellet (Innovative Research of America) was implanted. The skin was closed, and the mice were allowed to recover in a ThermoCare warmer. Sham animals underwent the same procedure without ligation around the aorta or implantation of a DOCA pellet. All experiments were approved by the University of Arizona Institutional Animal Care and Use Committee and followed the U.S. National Institutes of Health Using Animals in Intramural Research guidelines for animal use.

### Metformin

Metformin (Sigma-Aldrich) was added to the drinking water of adult TAC/DOCA mice (individually housed) 1 wk after surgery and was maintained for 5 wk. To get a correct dosage, mice were weighed and water intake was tracked, and metformin was dissolved in water to achieve a dose of 0.2 mg/g BM/day. This is within the range of previous rodent studies (Senesi et al., 2016; Sun and Yang, 2017) and aligns with levels achieved in routine diabetes treatment (Chandel et al., 2016).

### Echocardiography

Echocardiography was performed as previously described (Radke et al., 2007; Mohammed et al., 2010; Bull et al., 2016). Mice were anesthetized under 1% isoflurane (USP Phoenix) in oxygen mixture, then placed in dorsal recumbence on a heated platform for echocardiography. Transthoracic echo images were obtained with a Vevo 2100 High Resolution Imaging System (Visual-Sonics) using the model MS550D scan head designed for murine cardiac imaging, and MS250 scan head was used to measure aortic flow velocity at the site of constricted aorta. Care was taken to avoid animal contact and excessive pressure that could induce bradycardia. Body temperature was maintained at 37°C. Imaging was performed at a depth setting of 1 cm. Images were collected and stored as a digital cine loop for offline calculations. Standard imaging planes, M-mode, Doppler, and functional calculations were obtained according to American Society of Echocardiography guidelines. The parasternal long axis view and mid wall cross-sectional view of the LV were used to guide calculations of percentage fractional shortening, percentage ejection fraction, and ventricular dimensions and volumes. In addition, the left atrial dimension was measured in the long-axis view directly below the aortic valve leaflets. Passive LV filling peak velocity, E (cm/s), and atrial contraction flow peak velocity, A (cm/s), were acquired from the images of mitral valve Doppler flow from tilted parasternal long-axis views. A sweep speed of 100 mm/s was used for M-mode and Doppler studies. The heart rates of animals during echocardiographic study were maintained in the range of 500–550 beats per minute for M-mode, 500–550 beats per minute for B-mode, and 400–500 beats per minute for Doppler studies. Measures of diastolic function, specifically the relationship between early and late filling (E/A ratio) and how quickly the flow velocity declines in early diastole (E-wave deceleration time), are reported.

### Pressure–volume analysis

In vivo pressure–volume analysis was performed in mice 5 wk after metformin/vehicle treatment (6 wk after TAC) using a SciSense Advantage Admittance Derived Volume Measurement System and 1.2F catheters with 4.5-mm electrode spacing (SciSense). Mice were anesthetized and ventilated with 1% isoflurane using an SAR-1000 Ventilator (CWE Inc.), and body was temperature maintained at 37°C using a MouseMonitor S platform (Indus Instruments). Mice were secured, and the abdomen was opened below the sternum. The apex of the LV was punctured using a 28-gauge needle, and the catheter was advanced into the LV. The inferior vena cava was located and occluded during a sigh (pause) in ventilation to acquire load-independent indexes. Data acquisition and analysis was performed in LabScribe2 (iWorx). Pressure–volume data were analyzed using a monoexponential fit (*P* = *Ae^βV^* + c) with the exponent (β) reported as the stiffness (Burkhoff et al., 2005).

### Treadmill exercise tolerance test

Mice were run on an indirect colorimetry treadmill (TSE Systems) before and 6 wk after TAC/DOCA procedure. Mice underwent a graded exercise tolerance test until exhaustion as follows (speed, duration, incline grade): 0 m/min, 3 min, 0°; 6 m/min, 2 min, 0°; 9 m/min, 2 min, 5°; 12 m/min, 2 min, 10°; 15 m/min, 2 min, 15°; 18 m/min, 1 min, 15°; 21 m/min, 1 min, 15°; 23 m/min, 1 min, 15°; and +1 m/min, each 1 min thereafter, 15°. Exhaustion was defined as the point at which mice maintained continuous contact with the shock grid for 5 s or 20 visits to the shock grid in a 1-min period. During testing, gas was collected continuously and analyzed every 5 s. PhenoMaster software (TSE Systems) recorded and calculated oxygen consumption (VO_2_), carbon dioxide consumption (VCO_2_), the respiratory exchange ratio (RER), distance run, and visits to the shock grid. The VO_2max_ was determined by the peak VO_2_ reached during this test when RER was >1.0. Maximum running speed was defined as the treadmill speed at which VO_2max_ was achieved. Mice failing to reach an RER >1.0 were excluded.

### Muscle mechanics

Mice were placed under isoflurane anesthesia and cervically dislocated, and the ribcage was rapidly opened to access the heart. 1 ml HEPES, pH 7.4 (in mM: NaCL, 133.5; KCl, 5; NaH2PO4, 1.2; MgSO4,1.2; HEPES, 10), solution containing 30 mM KCl and 3 mM 2,3-butanedione monoxime was injected into the LV through the apex, after which time the heart noticeably relaxed and ceased pumping. The heart was removed and the LV was isolated from the other chambers. The apex and base were removed from the LV leaving a cross-sectional slice ∼2 mm thick. The septum and right ventricular attachment regions were discarded, and the LV free wall tissue was placed in relaxing solution (in mM: 20 BES, 10 EGTA, 6.56 MgCl2, 5.88 NaATP, 1 DTT, 46.35 K-propionate, 15 creatine phosphate, pH 7.0). Endocardial fibers (apical to base orientation) were dissected and discarded. Once visualized, midmyocardial fibers (circumferential orientation) were carefully removed and skinned in fresh relaxing solution, pH 7.0, with 1% Triton X-100 (Pierce) overnight at 4°C and protease inhibitors (PMSF, 0.5 mM; leupeptin, 0.04 mM; E64, 0.01 mM), then washed for 1 h with relaxing solution. 1 ml Phosphatase inhibitor cocktail 2 (P5726; Sigma-Aldrich) was added into 100 ml relaxing solution. Immediately after the wash with relaxing solution, 150- to 250-µm (diameter) strips were dissected, and aluminum clips were placed on both ends of the preparation. The aluminum clips were attached to a strain gauge force transducer and high-speed length motor, and the preparation was submersed in relaxing solution. A laser diffraction system was used to measure the sarcomere length by taking advantage of Bragg’s law (dsinθ = mλ) to calculate sarcomere length (d) based on the wavelength (λ), peak order (m), and angle (θ). The length and cross-sectional area of the fiber were then measured in order to normalize measured forces to cross-sectional area. Once the fiber was mounted and measured, a stretch–hold–release protocol was used in which a skinned fiber dissected from the circumferentially aligned free wall was stretched to a given sarcomere length within the physiological range, held for 90 s, and subjected to a sinusoidal frequency sweep. Because cardiac muscle is viscoelastic, the sinusoidal frequency sweep was used to quantify the viscous and elastic moduli. Viscous and elastic moduli are defined as (σ/ε)sin(θ) and (σ/ε)cos(θ), respectively, where σ is stress, ε is strain, and θ is the phase shift. To quantify the relative contributions of titin and ECM, skinned muscle strips were extracted using first 0.6 M KCl in relaxing solution followed by 1.0 M KI in relaxing solution to depolymerize the thick and thin filaments, respectively, removing the anchoring points of titin but keeping ECM intact (Granzier and Irving, 1995; Wu et al., 2000). Repeating the same initial protocol after extraction allows for calculation of titin-based stiffness by subtracting the postextraction (ECM) curve from the preextraction (total) curve.

### Titin isoform expression

The LV tissues were flash frozen in liquid nitrogen and solubilized between glass pestles cooled in liquid nitrogen. Tissues were primed at −20°C for a minimum of 20 min, then suspended in 50% urea buffer ([in mol/liter] 8 Urea, 2 Thiourea, 0.05 Tris-HCl, 0.075 Dithiothreitol with 3% SDS, and 0.03% Bromophenol blue, pH 6.8) and 50% glycerol with protease inhibitors ([in mmol/liter] 0.04 E64, 0.16 Leupeptin, and 0.2 PMSF) at 60°C for 10 min. Then the samples were centrifuged at 13,000 rpm for 5 min, aliquoted, flash frozen in liquid nitrogen, and stored at −80°C (Lahmers et al., 2004). Using agarose gel electrophoresis, total titin, N2B titin, N2BA titin, and T2 were analyzed. Briefly, the solubilized samples were electrophoresed on 1% agarose gels using a vertical SDS-agarose gel system (Hoefer; Warren et al., 2003a,b). Gels were run at 15 mA per gel for 3 h and 20 min, then stained using Coomassie brilliant blue (Acros organics), scanned using a commercial scanner (Epson 800; Epson Corporation), and analyzed with One-D scan (Scanalytics Inc.). Each sample was loaded in a range of five volumes, and the integrated optical densities (IOD) of titin and myosin heavy chain were determined as a function of loading volume. The slope of the linear relationship between IOD and loading was obtained for each protein to quantify expression ratios (Hidalgo et al., 2009).

### Splicing analysis

The dual luciferase assay and cell viability assays have previously been described (Liss et al., 2018). In brief, human embryonic kidney (HEK293.EBNA) cells were seeded in a 96-well plate and transfected with splice reporter plus RBM20 or pcDNA3.1 control plasmid using PEI40. 24 h later, Metformin was applied at nine serial twofold dilutions starting at 20 µM, and luciferase activity was measured 60 h after transfection using the Dual-Luciferase Reporter Assay System (Promega). Ratios of firefly to renilla luciferase activity were normalized to the control (pcDNA3.1). Cell viability was measured 60 h after transfection using a resazurin-based staining of metabolically active cells (PrestoBlue; Life Technologies).

### Phosphorylation

Solubilized samples were run on a 0.8% agarose gel in a vertical gel electrophoresis chamber. Gels run at 15 mA per gel for 3 h and 20 min were then transferred onto PVDF membranes (Immobilon-FL; Millipore) using a semidry transfer unit (Trans-Blot Cell; Bio-Rad). Blots were then probed with primary antibodies (described below) followed with secondary antibodies conjugated with fluorescent dyes with infrared excitation spectra (Biotium Company). Blots were scanned using an Odyssey Infrared Imaging System (Li-COR Biosciences), and the images were analyzed using Li-COR software. Signal was normalized to total protein transferred quantified either by Ponceau S (Sigma) staining (S11878, S12022) or by primary antibody colabeling (S4010). Ponceau S scans were analyzed in One-D scan (Hidalgo et al., 2009). Phosphorylation of the S11878 (GL Biochem) and S12022 PKC (Genescript) sites in the PEVK element of titin was probed with primary phospho-specific antibodies that have been described previously (Hudson et al., 2011). Phosphorylation of the S4010 sites was probed via phospho-specific antibodies (GL Biochem). Because this antibody has not been described before, we present the following details. The p-S4010 was raised in rabbit against CEEGK(pS)LSFPLA and had a high titer when used in ELISA assays with the phospho-peptide and very low titer when used against the unphosphorylated version of the peptide (CEEGK(S)LSFPLA). To test the specificity of the pS4010 when used on full-length titin, skinned fibers from WT and N2B KO mice were used. The WT fibers were either left untreated, treated with PP1 to dephosphorylate titin, or treated with PKA to phosphorylate PKA sites on titin. Fibers were then solubilized, run on a 0.8% agarose gel, and transferred using the Biorad system as described previously (Hidalgo et al., 2009). Ideal phospho-specific (pS4010) antibodies would yield strong signal for the PKA treated fiber, moderate signal for the untreated assuming some basal phosphorylation level, no to low signal for the PP1 treated, and no signal in the N2B KO (used for control) since the S4010 site exists in the deleted portion of the protein. However, using the antibodies to probe the phosphorylation status of these fibers yielded nonspecific binding—in all samples, including the N2B KO. To increase the specificity of the antibody, the SulfoLink Immobilization Kit for Peptides (ThermoFisher) was used. Here, an affinity column was created by immobilizing the nonphosphorylated version of the peptide that was used to raise the antibody. The antibody likely contains both phospho- and nonphospho-specific antibodies, and the phospho-specific fraction was removed in this step. The antibody was allowed to flow through the column with the nonphospho antibodies binding to the peptide allowing for collection of the phospho-specific fraction. In Western blots, nonspecific binding of the antibody was very low, as revealed by the minimal signal observed when using the antibody against the N2B KO lysate. Each blot was run with N2B KO samples in addition to experimental samples. Phosphorylation was reported relative to the N2B KO signal as this represents the remaining nonspecific background of the antibody. Western blots that are reported in this study were probed using the cleaned-up antibodies, and proteins were colabeled with the Z1Z2 titin antibody (Gregorio et al., 1998) to quantify total titin and normalize for loading differences (Warren et al., 2003b). Samples were run in triplicate, and an average of three technical replicates was taken as a biological sample.

### Statistical analysis

**Figure 1.**
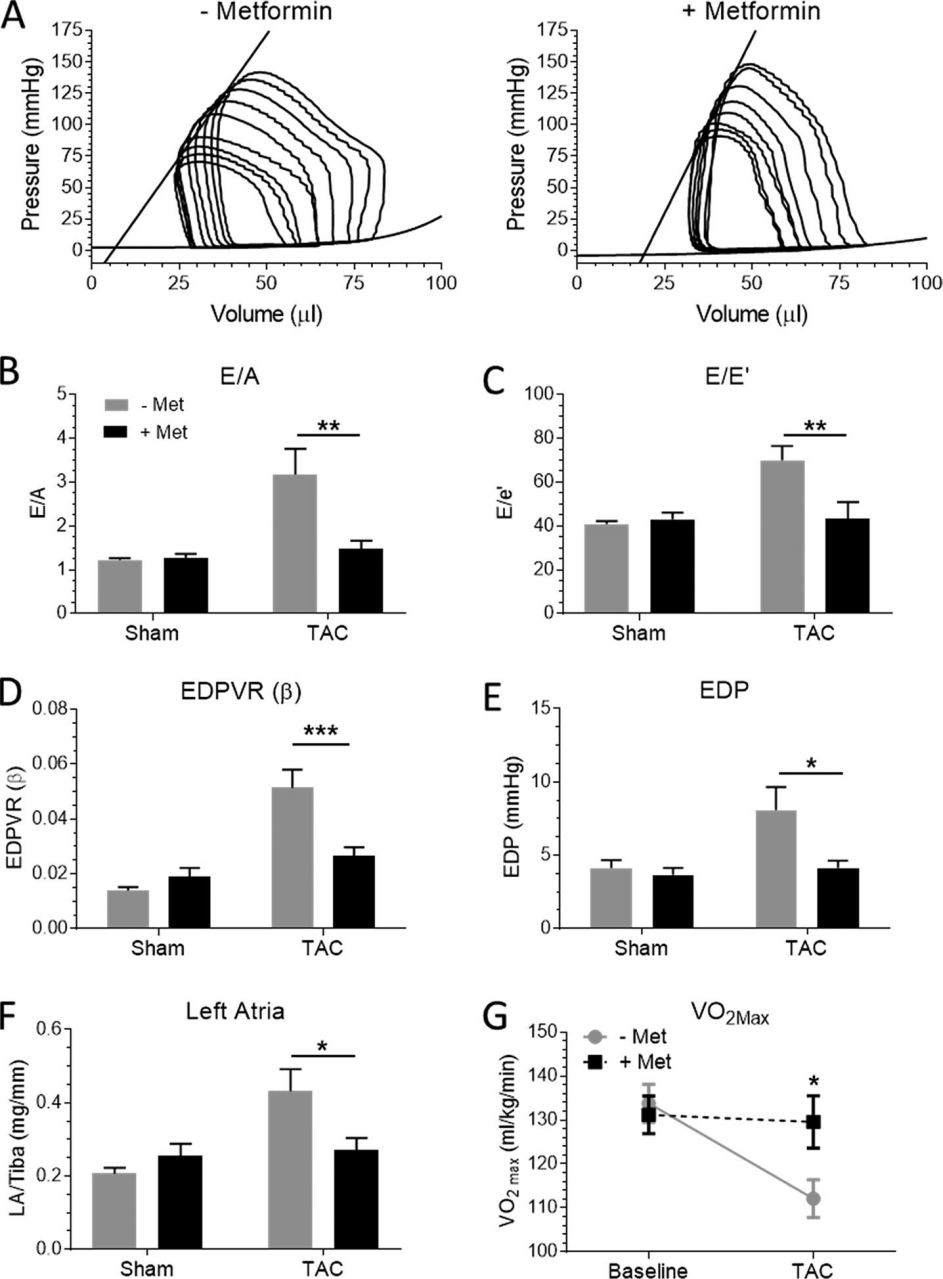
**Metformin improves diastolic function and exercise performance in TAC/DOCA WT mice.** Mice underwent a sham or TAC/DOCA procedure followed by treatment with vehicle or metformin 1 wk later. After 5 wk metformin treatment, diastolic function was assessed by echocardiography and pressure–volume (PV) analysis. **(A)** Representative PV loops of vehicle and metformin-treated TAC/DOCA mice. **(B–E)** After TAC/DOCA, metformin treatment attenuated the increase in E/A ratio (B), E/e′ ratio (C), β coefficient of the end-diastolic PV relation (EDPVR; D), and end-diastolic pressure (EDP; E). **(F)** The increase in left atria weight was attenuated by metformin. Exercise performance was assessed by treadmill testing with indirect calorimetry. **(G)** The TAC/DOCA mice demonstrated exercise intolerance with a reduced VO_2_max, which was preserved in the metformin-treated mice. *, P < 0.05; **, P < 0.01; ***, P < 0.001. *n* = 9 (sham –Met), 9 (sham +Met), 10 (TAC/DOCA –Met), and 8 (TAC/DOCA +Met). Error bars represent SEM.

**Figure 2.**
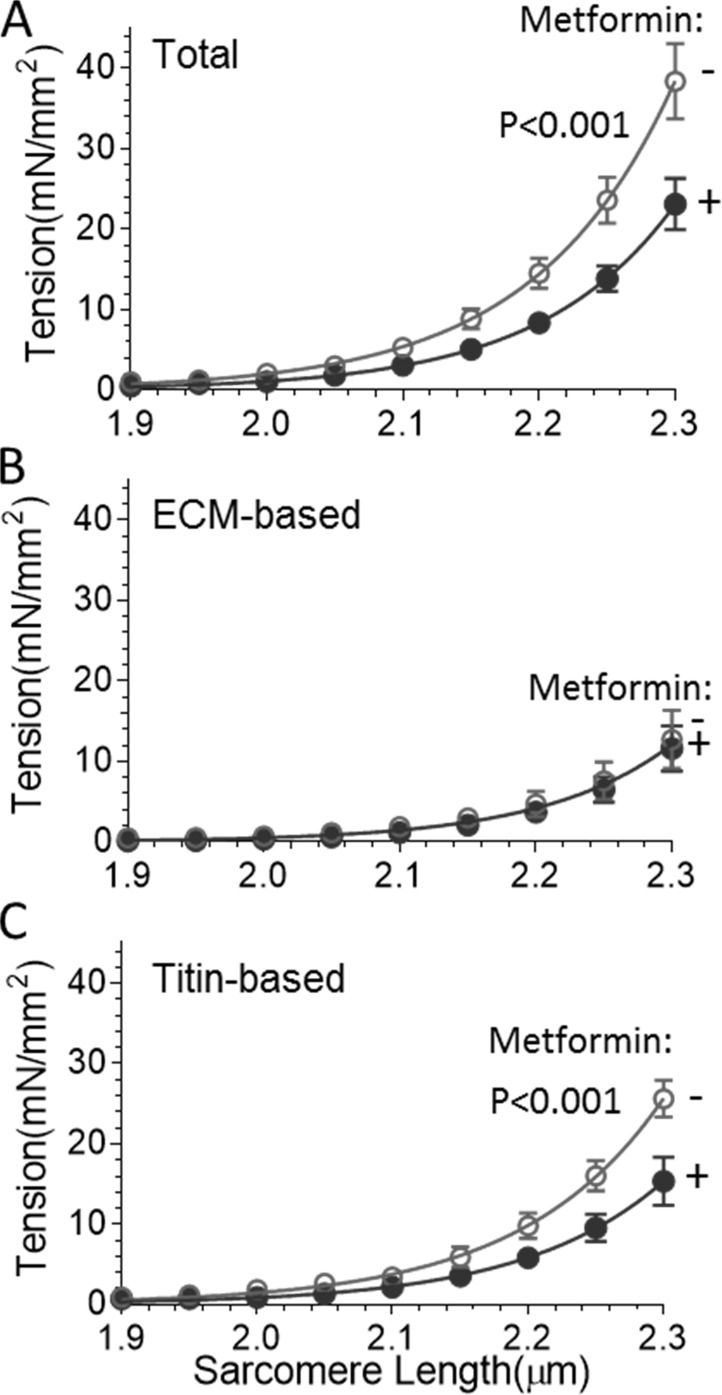
**Metformin reduces titin-based passive tension of LV muscle strips isolated from TAC/DOCA WT mice.** The TAC/DOCA mice without (*n* = 9) and with (*n* = 8) metformin treatment were studied. **(A)** Total passive tension was decreased in metformin-treated animals (P < 0.001). **(B)** ECM-based tension was unaffected by metformin (symbols overlap). **(C)** Titin-based tension was significantly (P < 0.001) reduced by metformin. Error bars represent SEM.

**Figure 3.**
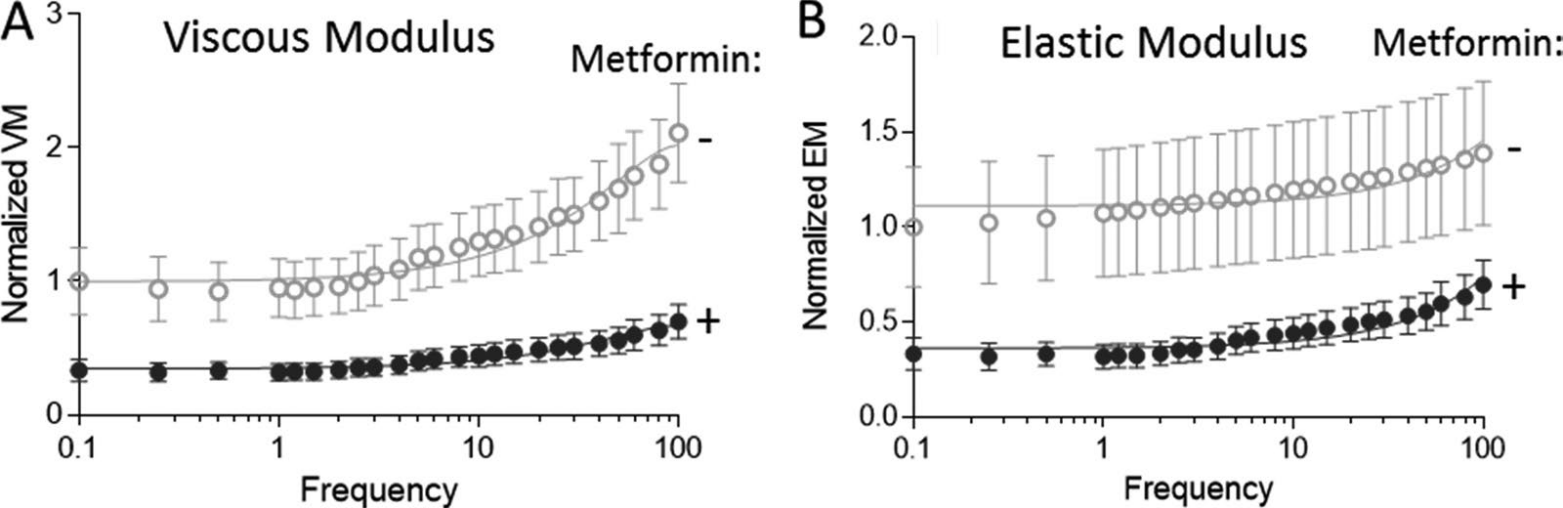
**Effect of metformin on viscous and elastic moduli of LV muscle strips isolated from TAC/DOCA WT mice.** Viscous moduli (VM) and elastic moduli (Emod) are obtained from a sinusoidal frequency sweep as the ratio of the stress over the strain times the sine (VM) or cosine (Emod) of the phase shift (Materials and methods section). Absolute VM and EMod are measured in mN/mm^2^; here values obtained at a sarcomere length of 2.3 µm on unextracted fibers are plotted normalized to the value of the untreated sample at the lowest frequency (this value is set to 1). **(A)** Viscous moduli were decreased in metformin-treated animals (P < 0.001). **(B)** Elastic moduli were decreased in metformin-treated animals (P < 0.001). Closed symbols indicate metformin-treated animals (*n* = 6); open symbols indicate no metformin treatment (*n* = 4). Error bars represent SEM.

**Figure 4.**
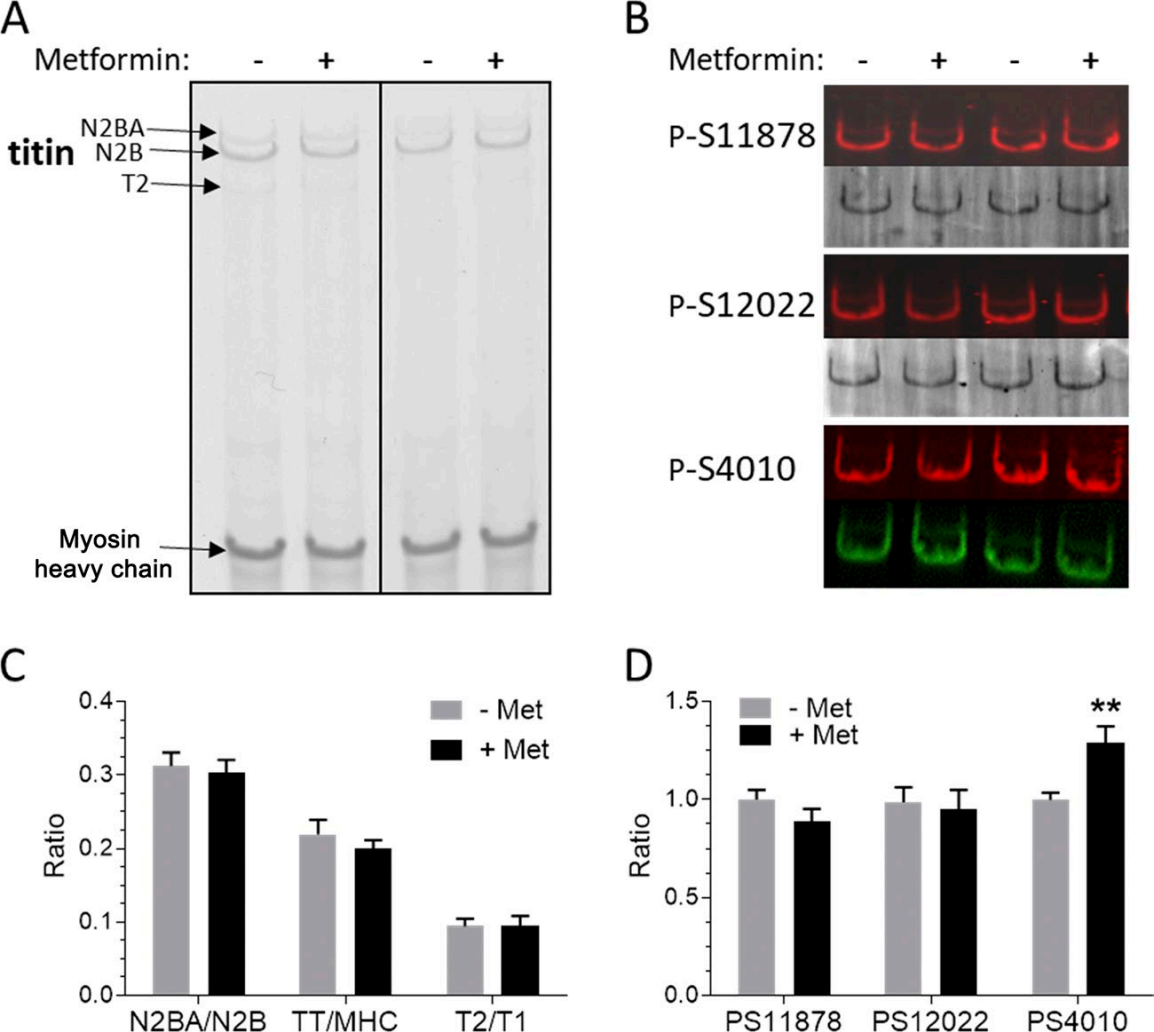
**Effect of metformin on protein expression and phosphorylation status of titin in TAC/DOCA mice**. **(A)** Sample titin agarose gels used to study the expression ratio of N2BA titin versus N2B titin, total titin (TT) versus myosin heavy chain expression, and the level of the degradation product T2 versus intact titin (T1, N2BA, and N2B isoform combined). **(B)** Sample Western blot (WB) results with antibodies that detect phosphorylated S11878 and S12022 in the PEVK (numbering based on the PEVK of the N2B isoform) and phosphorylated S4010 in the N2B element. Top image in each pair shows WB, and bottom image shows PonceauS stained membrane to normalize WB result for loading, except for p-S4010, where we double labeled with an antibody to titin’s N terminus (Z1Z2; green), and its signal was used for normalization (Materials and methods section). **(C)** Summarized protein expression results show that metformin does not affect titin expression levels. **(D)** There were no differences in phosphorylation of S11878 or S12022, but phosphorylation of S4010 was increased in the metformin-treated mice (*n* = 8). Error bars represent SEM.

**Figure 5.**
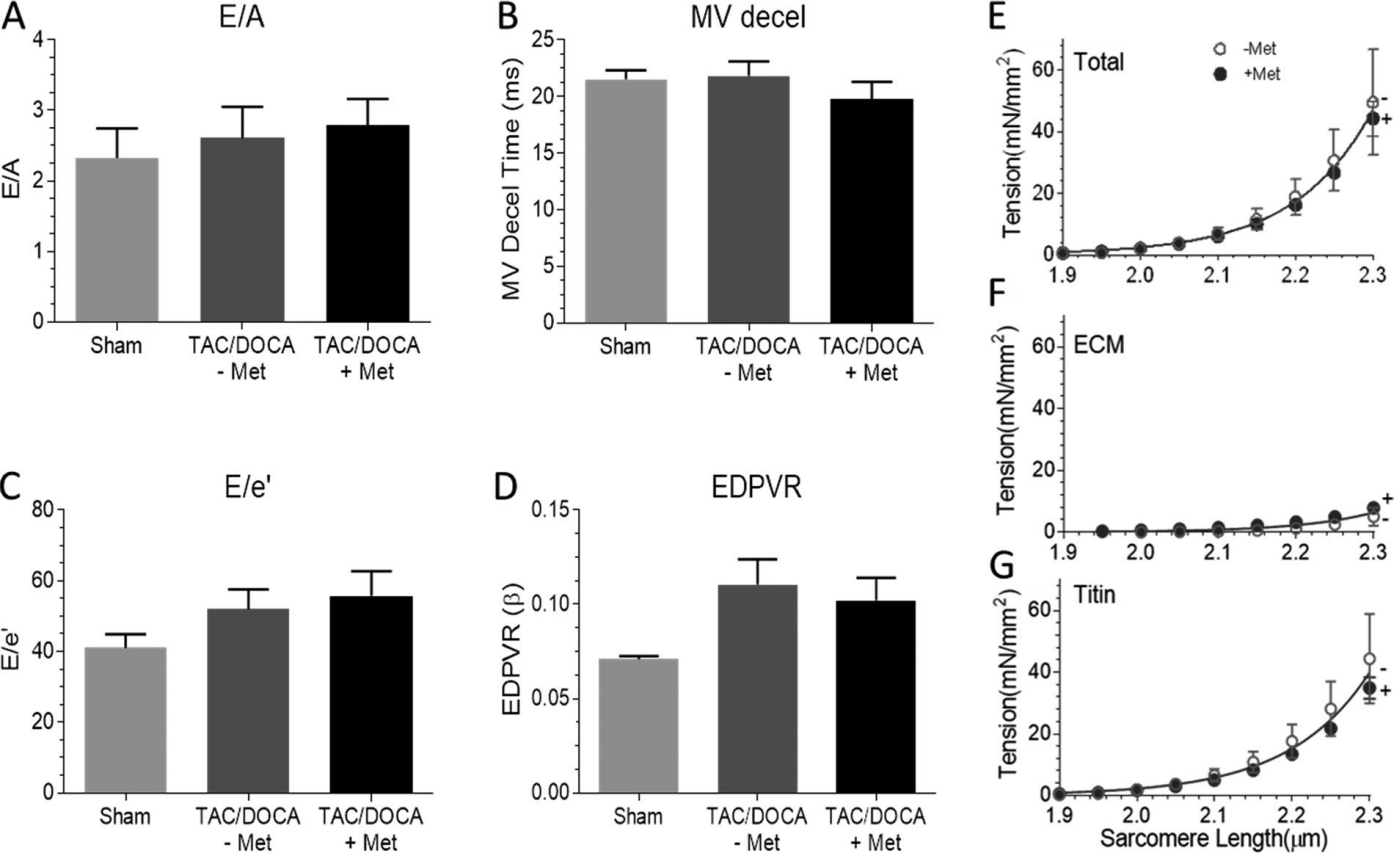
**Metformin does not affect diastolic function and LV passive tension in TAC/DOCA N2B KO mice. (A–C)** Echocardiography-derived diastolic function parameters from N2B KO mice with or without metformin treatment indicate no effect of metformin in N2B KO mice. **(D)** The LV stiffness assessed by pressure–volume loop analysis revealed no effect of metformin on the end-diastolic pressure–volume relation (EDPVR). **(E–G)** Total passive tension (E), ECM-based tension (F), and titin-based tension (G) measured in LV muscle strips from N2B KO mice were also unaffected by metformin. *n* = 4 (sham), 7 (TAC/DOCA –Met), and 6 (TAC/DOCA +Met). MV, mitral valve. Error bars represent SEM.

Data were analyzed (GraphPad Prism) using *t* test (Fig. 4), two-way ANOVA (Figs. 1 and 5, Fig. S2, and Tables S1–S3), or nonlinear regression (Figs. 2, 3, and 5, and Fig. S2) as appropriate. In the latter, a statistical analysis was performed to test whether the data sets were best fit by one or two equations. A nonlinear regression was performed with an extra sum-of-squares F test to determine whether there was evidence that the data sets differed from each other. The simpler model (i.e., single equation) was preferred for P values <0.05.

### Online supplemental material

The dual luciferase assay and cell viability assays conducted in HEK293.EBNA cells are shown in Fig. S1. Ratios of firefly to renilla luciferase activity were normalized to the control (pcDNA3.1). Cell viability was measured 60 h after transfection. Fig. S2 shows the effect of metformin on echo-based diastolic function and LV passive tension in N2B KO mice at baseline. Additional details are in the legends of Figs. S1 and S2. Tables S1–S3 show echocardiography and PV analysis data of HFpEF mice, N2B KO mice, and TAC/DOCA N2B KO mice, respectively.

## Results

### Effect of metformin on diastolic LV function of mice with HFpEF-like symptoms

To create an HFpEF-like state in the mouse, we induced a mineralocorticoid excess (DOCA pellet implantation) in the presence of chronic pressure overload (TAC surgery). The TAC/DOCA procedure results in concentric hypertrophy, exercise intolerance, fibrosis, and diastolic dysfunction, which overlaps with symptoms of HFpEF patients (Mohammed et al., 2010; Methawasin et al., 2016). Diastolic dysfunction of TAC/DOCA mice is evidenced 6 wk after surgery by altered diastolic filling parameters measured by Doppler echocardiography. Specifically, increased ratios of E to A and E to e′ and a contaminant increase in filling pressure were observed, suggesting increased LV chamber stiffness; pressure–volume analysis confirmed an increased diastolic stiffness of the LV based on an increase in the diastolic stiffness coefficient (β) of the end diastolic pressure volume relation (Table S1 and Fig. 1, A–D). The increased filling pressure is supported by the increased left atria weight in TAC/DOCA mice (Fig. 1 F). Mice treated with metformin for 5 wk after the TAC/DOCA procedure demonstrated that metformin did not affect the increased afterload experienced by TAC/DOCA hearts or the LV hypertrophy response, because end systolic LV pressures were comparable between TAC/DOCA groups (Table S1). Additionally, LV wall thickness and tissue weights were increased after TAC/DOCA, but metformin treatment had no effect on these values (Table S1). However, mice treated with metformin demonstrated preserved diastolic function similar to sham control groups, and left atrial weight and end diastolic pressure were now normal (Fig. 1, A–F). Exercise intolerance is a key symptom of HFpEF, which is captured in the TAC/DOCA model. After TAC/DOCA, vehicle-treated mice demonstrated a reduction in VO_2max_ during exercise tolerance testing, and metformin treatment preserved VO_2_max (Fig. 1 G).

### Effect of metformin on LV muscle stiffness of mice with HFpEF-like symptoms

To gain mechanistic insights into the effect of metformin on diastolic function, we studied muscle strips isolated from the midregion of the LV free wall. Muscle strips were demembranated and bathed in relaxing solution, making it possible to study their passive material properties as the muscle strips were stretched from their slack sarcomere length (∼1.9 µm) to a sarcomere length of 2.3 µm. Passive tension was measured before and after thick and thin filament extraction (Materials and methods section). This removes the anchors of titin in the sarcomeres, leaving only the ECM-based tension component (Granzier and Irving, 1995). The titin-based tension is determined from the difference between total passive tension (before extraction) and ECM-based passive tension (after extraction). Total passive tension was significantly lower in metformin-treated TAC/DOCA mice compared with untreated mice (Fig. 2 A). The ECM-based tension was found to be unaffected (Fig. 2 B). Subtracting ECM-based tension from the total tension revealed a statistically significant decrease in titin-based passive tension in metformin-treated TAC/DOCA mice (Fig. 2 C). To gain insights in the dynamic stiffness of the myocardium, we also performed a small-amplitude sinusoidal oscillation study at a range of frequencies (0.25–100 Hz) and measured both the viscous and elastic moduli. Both viscous (Fig. 3 A) and elastic moduli (Fig. 3 B) were significantly lower in metformin-treated TAC/DOCA mice compared with untreated TAC/DOCA mice. Thus, metformin lowers the stiffness of heart muscle under static and dynamic conditions, and the effect is derived from titin.

### Effect of metformin on titin expression and phosphorylation

To determine the molecular basis of the metformin-induced reduction in titin-based passive stiffness, titin expression and phosphorylation status were investigated. Using agarose gel electrophoresis (Warren et al., 2003b) we determined total titin (expressed relative to myosin heavy chain), N2B titin, N2BA titin, and the titin degradation product T2 (Fig. 4 A). Passive stiffness may be reduced because of the reduction in the total expression level of titin, a shift in the titin isoform expression ratio from the stiff N2B isoform to the more compliant N2BA isoform, or degradation of titin (T2 level). However, there were no changes in total titin, N2BA/N2B, or T2/total titin (Fig. 4, A and C). We also used a dual luciferase splice reporter assay that builds on the titin splice factor RBM20 and studied the effect of metformin on titin splicing and cell viability in HEK293 cells (for details, see Liss et al., 2018). In line with the protein expression data, there was no effect of metformin on titin splicing (Fig. S1). Metformin was well tolerated but did not affect the splice reporter readout.

In addition to changes in expression of titin, the stiffness of titin can also be altered through a change in the phosphorylation level of titin’s PEVK and N2B spring elements. Phosphorylation of the S11878 and S12022 sites in the PEVK element of titin that are targets of PKCα (Hidalgo et al., 2009) and phosphorylation of the S4010 site in the N2B element of titin that is a PKA target (Yamasaki et al., 2002) were studied via Western blot analysis with phospho-specific antibodies (Fig. 4 B). No metformin effects were found in the phosphorylation level of the two PEVK sites in metformin-treated mice, but phosphorylation of S4010 was significantly increased (Fig. 4 D). Because elevated phosphorylation of PKA sites in titin has been shown to lower passive stiffness, the increased phosphorylation of S4010 is likely to contribute to the reduction in diastolic stiffness in metformin-treated TAC/DOCA mice.

### Effect of metformin on diastolic function of N2B KO mice

To further study the importance of titin’s N2B element in the beneficial effect of metformin on diastolic function of TAC/DOCA mice, we investigated a mouse model in which the N2B element of titin has been deleted (the N2B KO). The N2B KO mice have increased diastolic stiffness due to the higher strain on the remaining titin spring elements (Radke et al., 2007). We tested whether metformin was able to improve diastolic function and titin-based passive stiffness in N2B KO mice. Echocardiography showed no effect of metformin on diastolic function in either WT or N2B KO mice under baseline conditions, while N2B KO mice demonstrated diastolic dysfunction compared with WT mice under baseline conditions (Table S2 and Fig. S2, A–C). Passive tension measurements of LV wall muscle were also performed in the N2B KO. As above, muscle strips dissected from the LV free wall were studied to determine the contribution of the ECM and titin to myocardial stiffness. Although total and titin-based passive stiffness were higher in untreated N2B KO mice than the WT mice, total passive stiffness (Fig. S2 D), ECM passive stiffness (Fig. S2 E), and titin passive stiffness (Fig. S2) were not significantly different in metformin-treated N2B KO mice.

The lack of a response to metformin in the N2B KO mice under baseline conditions could be explained by the absence of certain reversible changes specific to the TAC/DOCA pathology. Therefore, a group of N2B KO mice underwent TAC/DOCA surgery and were then treated with metformin in order to determine whether the N2B element plays a critical role in mediating the metformin response. The TAC/DOCA numerically worsened the diastolic function assessed by both pressure–volume analysis and echocardiography in N2B KO mice; however, metformin treatment caused no differences in diastolic parameters in TAC/DOCA N2B KO mice (Table S3 and Fig. 5, A–D). Additionally, total, ECM-based and titin-based passive stiffness were also unaffected (Fig. 5, E–G). Thus, the N2B element appears to be essential for the effect of metformin on diastolic function.

## Discussion

This study revealed that metformin, a commonly used oral drug for the treatment of type II diabetes, ameliorates diastolic LV chamber stiffness in mice with HFpEF-like symptoms by inducing a reduction in titin-based stiffness. This finding supports the importance of titin for LV diastolic stiffness and adds to previous work in which titin was made either stiffer (Radke et al., 2007; Chung et al., 2013; Granzier et al., 2014) or more compliant (Methawasin et al., 2014; Hinze et al., 2016) and that revealed that LV diastolic stiffness was altered accordingly. Although many other factors contribute to diastolic stiffness (speed of calcium removal from the cytoplasm, cross-bridge detachment kinetics, ECM stiffness, etc. [Kass et al., 2004]), titin appears to be a dominant factor. This improvement in diastolic function is associated with improved exercise capacity and VO_2_ max, suggesting metformin preserves cardiac reserve and performance during exercise. Metformin had no effect when administered to mice that lack the N2B element of titin, indicating that the N2B element is the downstream target within titin. The N2B element is a molecular spring within titin’s I-band region that extends as the sarcomere is stretched and contributes to the unique passive force–sarcomere length relation of cardiac myocytes (Helmes et al., 1999). The N2B element is cardiac-specific and is found in both adult cardiac titin isoforms, the short and therefore stiff N2B isoform and the longer and more compliant N2BA isoform (Bang et al., 2001). The N2B element is a hot spot for posttranslational modifications, with ERK1/2, CaMKIIδ, PKA, and PKG all targeting this element (Hidalgo and Granzier, 2013). Single-molecule and single-cell studies have shown that the functional result of phosphorylating the N2B element is an increase in compliance of the N2B element and a reduction in passive force (Yamasaki et al., 2002; Perkin et al., 2015). Thus, the increased phosphorylation level of S4010, a PKA site in the N2B element that was found in metformin-treated HFpEF mice, suggests that this effect contributes to the reduction in diastolic stiffness. Our finding that a PKA site within titin has elevated phosphorylation after metformin treatment is consistent with a recent study demonstrating that PKA activity is significantly elevated in metformin-treated cardiac myocytes, an effect mediated by AMP activated protein kinase (Kobashigawa et al., 2014).

In addition to lowering stiffness by PKA phosphorylation, titin’s stiffness is also modulated by other pathways. Recent work by Hopf et al. (2018) identified signaling pathways involved in regulating titin phosphorylation in response to insulin and metformin, which include PKG, ERK, and PKC pathways. In agreement with our findings, they were also able to demonstrate an increase in phosphorylation of the S4010 site after metformin treatment. Presently, it cannot be ruled out that additional modifications link metformin to a reduction in titin-based stiffness. Although it is unlikely that phosphorylation of a single residue within the N2B element of titin can fully explain the beneficial effects of metformin in this HFpEF-like model, the findings presented here suggest the N2B element is a critical region. Although no differences in overall titin protein expression were observed, changes in additional phosphorylation sites or oxidative changes within the N2B element may occur. One such possible modification is the oxidation of cysteines within the N2B element causing disulfide bonds, leading to an increase in titin stiffness (Nedrud et al., 2011). If fewer disulfide bonds were to exist in metformin-treated mice, this would add to the PKA-induced passive force reduction. Whether such changes exist within the N2B element of titin that respond to metformin treatment requires future research.

### Clinical implication

Several recent studies on biopsies from patients with HFpEF have revealed hypo-phosphorylation of a PKA site located in the N2B element of human titin (Borbély et al., 2009a,b; Zile et al., 2015) and activation of PKA upon administering metformin would be expected to increase phosphorylation of PKA sites on titin in patients as well. Thus, the present findings might be clinically relevant. The effect of PKA phosphorylation on passive force has been shown to be titin isoform dependent (Fukuda et al., 2005) with the largest effect detected in myocytes that express high levels of N2B titin and the smallest effect in myocytes that express high N2BA titin levels (changes in the compliance of the N2B element are attenuated by the longer tandem Ig and PEVK elements in N2BA titin; for details, see Fukuda et al., 2005). The expression ratio of the adult titin isoforms in the LV varies in different species (Cazorla et al., 2000), but the differences between HFpEF-like mice and HFpEF patients are relatively modest: ∼70% of total titin is N2B titin in the mouse (this study) and ∼65% in patients (Zile et al., 2015). Based on data in Fukuda et al. (2005), it can be estimated that the metformin-induced reduction in stiffness in the human ventricle would be ∼10% less than in the mouse and thus should be clearly detectable. In line with this expectation, phosphorylating skinned cardiomyocytes isolated from HFpEF patients by exogenously adding PKA has been shown to significantly decrease passive stiffness (Borbély et al., 2009a). We conclude that metformin might be effective in lowering titin-based passive stiffness in HFpEF patients and improving diastolic function.

Although the effect of metformin on diastolic function per se has not been investigated in HFpEF patients, a number of clinical studies have investigated the correlation between metformin and heart failure and reported positive correlations on mortality (Miles et al., 2014). Incident heart failure is reduced in people with type II diabetes on metformin (Nichols et al., 2005; Pantalone et al., 2009), and an analysis evaluating ∼20,000 people with diabetes in the REACH (REduction of Atherothrombosis for Continued Health) registry reported a 31% lower heart failure mortality rate in those patients on metformin compared with those not on metformin (Roussel et al., 2010). Although the work presented here demonstrates a benefit of metformin in improving passive stiffness in HFpEF-like mouse model, HFpEF patients often present with both increased passive stiffness and impaired active relaxation. The TAC/DOCA model shows only a small increase in the time constant for isovolumetric relaxation, Tau, which was unaffected by metformin treatment. Active relaxation and the effect of metformin treatment was not further evaluated due to minimal impairment of relaxation in this model; however, a retrospective cohort study of diabetes patients found a positive relationship between the use of metformin and improved LV relaxation (Andersson et al., 2010). These correlations support the hypothesis that metformin has a positive effect on diastolic function and might be effective in HFpEF patients. Clearly, studies are now required that focus specifically on the diastolic effect of metformin in HFpEF patients.

Pharmacological approaches for treating HFpEF are of particular interest because HFpEF therapies are lacking despite several large randomized clinical trials. A recent clinical trial (RELAX) sought to increase titin’s compliance through enhancing PKG activity using a phosphodiestesrase-5 inhibitor (sildenafil); however, exercise capacity and clinical outcomes were similar to placebo (Redfield et al., 2012, 2013) possibly due to the fact that sildenafil levels were associated with minimal increases in plasma cGMP (Redfield et al., 2013). The neutral outcome of the RELAX trial does not diminish the importance of a titin-based approach; titin remains an interesting target for diastolic stiffness–based therapies because it is a major determinant of diastolic function and has multiple well-established biological pathways for altering its stiffness, including the PKA pathways targeted by metformin. Recent work by Methawasin et al. (2016), Bull et al. (2016), and suggest that an alternative for improving diastolic function is inactivating the titin splicing RBM20. Genetically inactivating RBM20 causes the expression of supercompliant N2BA-type titin isoforms and improves diastolic function in mice with pathological diastolic stiffening (Methawasin et al., 2016). However, genetic targeting is relatively straightforward in the mouse but is very difficult to achieve in humans, and currently, no pharmaceuticals exist that can target RBM20. A clear advantage of lowering stiffness with metformin is that metformin is an existing drug with a known safety profile. In addition, in normoglycemic persons, metformin does not induce hypoglycemia (Kalra et al., 2013). Taking advantage of titin phosphorylation to modulate stiffness is of particular interest given that phosphorylation is known to be dysregulated in HFpEF patients (Borbély et al., 2009a; Zile et al., 2015). Considering the diastolic benefits demonstrated in the mouse model and the correlations between metformin treatment and LV function in human populations, we conclude that metformin is a potential therapy for patients with HFpEF. The use of an approved, well-tolerated drug such as metformin to target titin stiffness presents a unique opportunity for immediate translation.

In summary, metformin lowers LV diastolic stiffness in mice exhibiting an HFpEF-like phenotype, and the effect can be explained by a reduction in titin-based passive stiffness. The N2B element within cardiac titin is a downstream metformin target, and the mechanism includes phosphorylation of PKA sites within the N2B element. This makes metformin and titin-based modifications an exciting therapeutic avenue in treating diastolic dysfunction and HFpEF.

## Supplementary Material

Supplemental Materials (PDF)

Tables S1-S3 (XLS)
