# Supplementary material for: Metformin improves diastolic function in an HFpEF-like mouse model by increasing titin compliance
Source: J Gen Physiol. 2019 Jan 7;151(1):42–52. doi: 10.1085/jgp.201812259 (PMC6314384; doi:10.1085/jgp.201812259)
Supplement: Supplemental Materials (PDF) [file JGP_201812259_sm.pdf]

Supplemental material

Slater et al., https://doi.org/10.1085/jgp.201812259

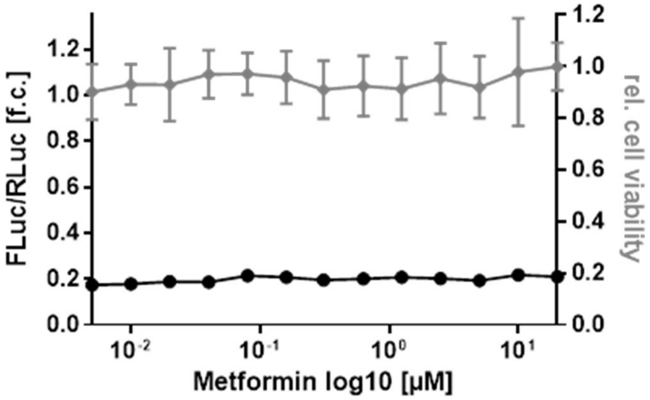

Figure S1. **Effect of metformin on RBM20-dependent titin exon inclusion.** 293 cells cotransfected with the dual luciferase reporter and the RBM20 expression plasmid exclude the alternative titin exon resulting in firefly to renilla luciferase ratios (FLuc/RLuc) of 0.2, independent of the amount of metformin added. Cell viability is also unaffected by metformin. Error bars represent SEM.

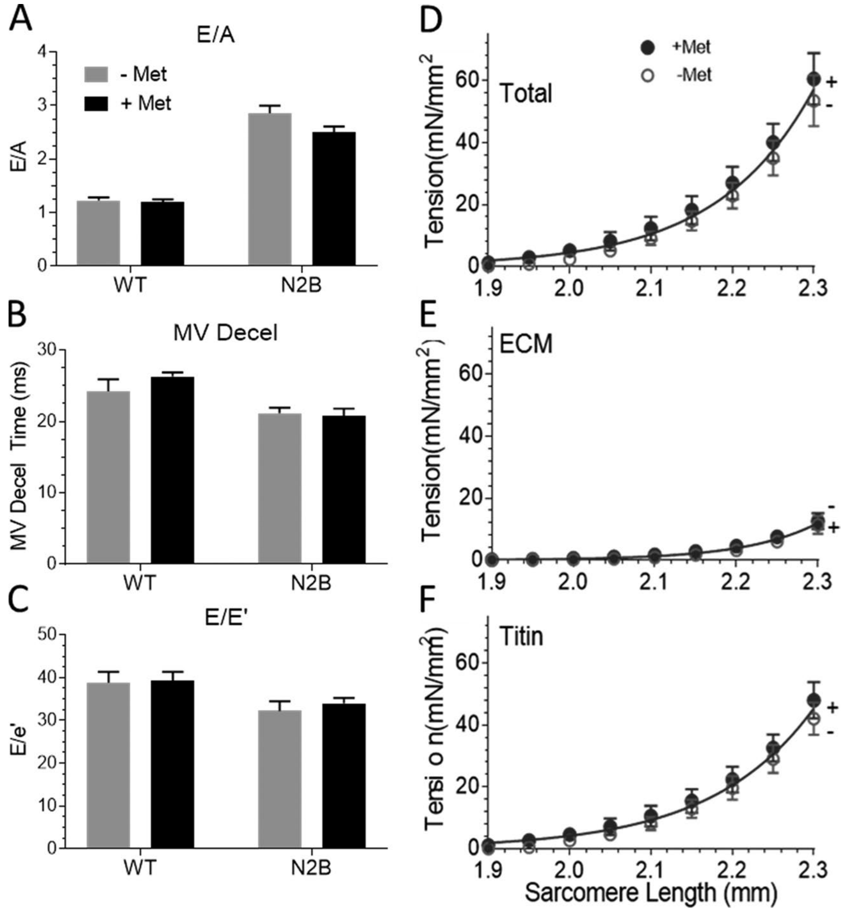

Figure S2. **Metformin does not affect echo-based diastolic function and LV passive tension in N2B KO mice at baseline. (A–C)** Echocardiography-derived diastolic function parameters from WT and N2B KO mice with or without metformin treatment indicate no effect of metformin under baseline conditions in WT or N2B KO mice. **(D–F)** Total passive tension (D), ECM-based tension (E), and titin-based tension (F) measured in LV muscle strips from N2B KO mice were unaffected by metformin treatment. *n* = 4 (sham −Met), 5 (sham +Met), 7 (TAC/DOCA −Met), and 7 (TAC/DOCA +Met). MV, mitral valve. Error bars represent SEM.

Tables S1–S3 are provided as an Excel file. Table S1 shows echocardiography (top) and pressure–volume analysis (bottom) of HFpEF mice with no treatment (-) or with metformin treatment (+). Abbreviations are as follows: s, systolic; d, diastolic; LV, left ventricle; LVID, LV internal dimension; LA, left atrium; MV, mitral valve; dP/dt max, maximal rate of pressure increase; dP/dt min, maximal rate of pressure decreases; Ea, effective arterial elastance; EDP, end-diastolic pressure; EDPVR, end-diastolic pressure–volume relation; EDV, end-diastolic volume; Ees, end-systolic elastance; EF, ejection fraction; ESP, end-systolic pressure; ESV, end-systolic volume; PRSW, preload recruitable stroke work; SV, stroke volume; and tau Logistic, left ventricular relaxation–time constant. *, P < 0.05 vs. sham control; **, P < 0.01 vs. sham control; #, P < 0.05 vs. TAC/DOCA without (-) metformin; and ##, P < 0.01 vs. TAC/DOCA without (-) metformin. Table S2 shows echocardiography parameters of N2B KO mice with no treatment (-) or with metformin treatment (+). Abbreviations are as follows: s, systolic; d, diastolic; LV, left ventricle; LVID, LV internal dimension; WT, LV wall thickness; LA, left atrium; and MV, mitral valve. *, P < 0.05 vs. WT control; ****, P < 0.0001 vs. WT control; ##, P < 0.01 vs. without (-) metformin. Table S3 shows echocardiography (top) and pressure–volume analysis (bottom) of TAC/DOCA N2B KO mice with no treatment (-) or with metformin treatment (+). Abbreviations are as follows: s, systolic; d, diastolic; LV, left ventricle; LVID, LV internal dimension; LA, left atrium; MV, mitral valve; dP/dt max, indicates maximal rate of pressure increase; dP/dt min, maximal rate of pressure decreases; Ea, effective arterial elastance; EDP, end-diastolic pressure; EDPVR, end-diastolic pressure–volume relation; EDV, end-diastolic volume; Ees, end-systolic elastance; EF, ejection fraction; ESP, end-systolic pressure; ESV, end-systolic volume; SV, stroke volume; tau Logistic, left ventricular relaxation–time constant. *, P < 0.05 vs. sham without (-) metformin; #, P < 0.05 vs. TAC without (-) metformin.
